# Comparative mRNA and MicroRNA Profiling during Acute Myocardial Infarction Induced by Coronary Occlusion and Ablation Radio-Frequency Currents

**DOI:** 10.3389/fphys.2016.00565

**Published:** 2016-11-25

**Authors:** Eduardo T. Santana, Regiane dos Santos Feliciano, Andrey J. Serra, Eduardo Brigidio, Ednei L. Antonio, Paulo J. F. Tucci, Lubov Nathanson, Mariana Morris, José A. Silva

**Affiliations:** ^1^Rehabilitation Department, Universidade Nove de JulhoSão Paulo, Brazil; ^2^Biophotonics Department, Universidade Nove de JulhoSão Paulo, Brazil; ^3^Medicine Department, Universidade Nove de JulhoSão Paulo, Brazil; ^4^Cardiac Physiology Department, Universidade Federal de São PauloSão Paulo, Brazil; ^5^Institute for Neuro-Immune Medicine, Nova Southeastern UniversityFort Lauderdale, FL, USA

**Keywords:** myocardial infarction, RNA, microRNAs, rats, Wistar, methods

## Abstract

The ligation of the left anterior descending coronary artery is the most commonly used experimental model to induce myocardial infarction (MI) in rodents. A high mortality in the acute phase and the heterogeneity of the size of the MI obtained are drawbacks recognized in this model. In an attempt to solve the problem, our group recently developed a new MI experimental model which is based on application of myocardial ablation radio-frequency currents (AB-RF) that yielded MI with homogeneous sizes and significantly reduce acute mortality. In addition, cardiac structural, and functional changes aroused by AB-RF were similar to those seen in animals with MI induced by coronary artery ligation. Herein, we compared mRNA expression of genes that govern post-MI milieu in occlusion and ablation models. We analyzed 48 mRNAs expressions of nine different signal transduction pathways (cell survival and metabolism signs, matrix extracellular, cell cycle, oxidative stress, apoptosis, calcium signaling, hypertrophy markers, angiogenesis, and inflammation) in rat left ventricle 1 week after MI generated by both coronary occlusion and AB-RF. Furthermore, high-throughput miRNA analysis was also assessed in both MI procedures. Interestingly, mRNA expression levels and miRNA expressions showed strong similarities between both models after MI, with few specificities in each model, activating similar signal transduction pathways. To our knowledge, this is the first comparison of genomic alterations of mRNA and miRNA contents after two different MI procedures and identifies key signaling regulators modulating the pathophysiology of these two models that might culminate in heart failure. Furthermore, these analyses may contribute with the current knowledge concerning transcriptional and post-transcriptional changes of AB-RF protocol, arising as an alternative and effective MI method that reproduces most changes seem in coronary occlusion.

## Introduction

The ligation of the left anterior descending coronary artery is a classical experimental model to induce myocardial infarction (MI) in rodents. However, this method showed a high mortality rate in the acute phase post-MI and large heterogeneity of the MI size that are recognized drawbacks of this model. Antonio et al. (2009) developed a new MI experimental protocol, which is based on application of ablation radio-frequency currents (AB-RF) in the myocardium. This technique, initially used as a heart failure (HF) method, yielded MI with homogeneous sizes, and significantly reduces acute mortality rate. Although cardiac structural and functional changes aroused by AB-RF were similar to those seen in animals with MI induced by coronary artery ligation (Antonio et al., 2009), extensive comparison of molecular signature of these models is yet unknown.

There are strong evidences of several upregulated, downregulated, or stable mRNA expression during MI and HF progression. Many authors assessed an expression pattern in different MI phases and issues as cardiomyocytes responses to remodeling were addressed in different studies, focusing in differentially expressed genes (Dhalla et al., 2012; Sofia et al., 2014; Katz et al., 2016; Kirk and Cingolani, 2016). These changes may occur at multiple levels, including in transcription regulation, epigenetic processes, mRNA stability, and more. MicroRNAs (miRs), a specific group of 18–24 nucleotides non-coding RNAs are compelling regulators that can both destabilizing and inhibiting translation of mRNAs (Kukreja et al., 2011).

To compare these two MI protocols, we performed the evaluation of mRNA expression of nine different signal transduction pathways (cell survival, cell cycle, oxidative stress, apoptosis, molecules related to calcium signaling, cell growth factors, transcription factors, angiogenesis, and inflammation) in rat left ventricle with MI generated by experimental protocols of coronary occlusion and AB-RF. Therefore, along with identification of differentially expressed genes, we performed a large-scale microRNA identification in these MI protocols. The mRNA and microRNA transcriptomes analyses may help to draw feasible molecular mechanisms associated with a particular MI model that seldom would have functional significance alone. Thus, AB-RF due its lack of complexity might be an effective and fast method to achieve MI.

## Methods

### Animals

Sixty-five male Wistar rats (~230 g) were used in the present work. The animals were cared for in compliance with the Principles of Laboratory Animal Care, as formulated by the National Institutes of Health (National Institutes of Health publication no. 96-23, revised in 1996). The protocol was approved by the Ethics Committee for Animal Care and Use at the Universidade Nove de Julho, in Sao Paulo, Brazil (034/2012). The animals were randomized into three groups: animals that undergo to left coronary descendent artery ligation (Occlusion, *n* = 37); animals that undergo to left ventricle RF ablation (Ablation, *n* = 21); and animals that had thoracotomy but neither occlusion or ablation were performed (Sham, *n* = 7).

### Anterior descending coronary artery ligation

This MI method was based on work by Johns and Olson (1954), with minor adaptations. This technique is being routinely applied to rat in our laboratory (Antonio et al., 2009; Manchini et al., 2014). Rats were anesthetized with 4% halothane inhalation, intubated and mechanically ventilated with positive pressure in rodent ventilator (Harvard Model 683, Holliston, MA, USA). After trichotomy, lateral thoracotomy was performed at the place where the heart impacts on palpation. With the animal in the supine position was made 2 cm incision in the skin and dilatation of the pectoral and intercostal muscles with the help of Kelly curved forceps. After dilatation of the intercostal muscles, the ribs were isolated with the help of Kelly forceps and retractor Stevenson adapted. Then it was held a pericardiotomy and exposure of the heart to visualize the anterior descending coronary artery (ADCA).

For “MI generation, ADCA was occluded to ~3 mm from the origin of the aorta through 5.0 nylon suture. After being checked the results of the suture, the retractor was removed, lung hyperinflation is promoted and the thorax was closed by purse string suture previously prepared around the incision edges. Postoperative care as analgesia (meperidine, 20 mg/kg, SC) and search for signs of anorexia, fever, vomiting, or abnormal respiration were conducted in all experimental animals.

### Radio-frequency ablation

Under 4% halothane anesthesia and immobilization in the supine decubitus position, the left thoracotomy was performed in the fourth intercostal space. The ribs were separated by retractors. After pericardium opening, the electrode (forceps) was placed in position to gently embrace the heart, and the catheter tip was placed on the LV anterolateral wall, perpendicularly to the tissue. AB-RF lesions (one ablation/rat) were achieved using a modified unipolar mode, following the procedure used by Antonio et al. (2009) and Dos Santos et al. (2013). Briefly, a custom-made catheter with a single electrode located at its tip was used to deliver RF energy against an indifferent electrode with a large area. The catheter tip was a single aluminum dome-shaped electrode (similar to a round domed screw head), 4.5 mm in diameter and 4.0 mm in length. This electrode was connected to an electrically-insulated flexible coaxial cable that was able to deliver very high frequency currents. A copper plate (14.6 mm) was located at the posterior aspect of the heart. Special steel forceps designed to support the heart during AB-RF was used as the indifferent electrode. The distal end of the forceps took the shape of 2 small shells (0.9 cm in diameter). Given the large surface of these shells compared with the rat heart, energy could be delivered to the myocardium without a significant rise in impedance. Thus, the cable was connected to the proximal end of the forceps.

A commercially available RF generator (model TEB RF10; Tecnologia Eletronica Brasileira Ltda, Sao Paulo, Brazil) was used to create RF-Ab lesions. RF current (1000 KHz) was delivered at constant power (12 watts) for 12 s. Power and impedance was monitored during each application and the mean values were recorded. An automatic power output shut down was triggered if impedance exceeded 200 ohms. The damaged tissue was characterized by the presence of a clear white disk-shaped region of coagulation necrosis that appeared immediately after current application. Then, the heart was instantly returned to the thorax, pulmonary hyperinsufflation was performed, and a previously made pursestring suture was used to close the chest. A continuously monitored D2 electrocardiogram was performed during the procedure. Ventricular Ab protocol was always lower than 2 min and postoperative care was performed as stated previously.

### Transthoracic doppler echocardiograms

Animals from occlusion and ablation groups undergo to ECHO examination to evaluate the infarct size 3 days after MI induction using a HP SONOS 5500 (Philips Medical System, Andover, MA) with a 12-MHz transducer at a depth of 2 cm, according to previous reports (Antonio et al., 2009; Silva et al., 2014). Briefly, under 4% halothane anesthesia, 2-dimensional and M-mode images from the parasternal longitudinal, transverse, and apical views were obtained and recorded on a 0.5-inch videotape. The imaging analysis and measurements were performed offline. MI was detected by ECHO on the basis of subjective identification of akinesis or dyskinesis. Measurements of end-diastolic (LVAd) and end-systolic (LVAs) LV transverse areas were performed in the 3 transverse planes (basal, medium, and apical) and LV systolic function was estimated by the fractional area change (FAC: LVAd—LVAs/LVAd × 100). Diastolic function was assessed by calculating the peak E and A blood flow mitral velocities and the E/A ratio. Sample volume of the pulsed wave Doppler was positioned at the tips of the mitral valve leaflets in an apical 4-chamber view. Only animals bearing infarct sizes higher than 40% of LV in both procedures were included in the experimental groups. An experiment cardiologist made a blind analysis of all echocardiograms.

### mRNA quantification analysis

Animals were euthanized by decapitation 1 week after each MI protocols. An area from a remote MI site (septum) was collected from each animal and used for comparative messenger RNA expression using TaqMan microarray plates to identify mRNAs that were differentially expressed among the different groups of rat heart tissues; control, occlusion, and ablation MI samples.

Samples weighing between 0.2 and 0.5 g, were homogenized in Trizol® Reagent for extracting RNA according to manufacturer's instructions. Then, extraction was performed with equal volume mixture containing phenol-chloroform-isoamyl alcohol in a ratio of 25:24:1, followed by precipitation with 0.2 M sodium acetate and 2 volumes of absolute ethanol. The precipitated RNA was washed with 70% ethanol to eliminate the phenol and salt residues, and solubilized in DEPC-water. The total RNA extracted was treated with deoxyribonuclease 10 U RNase-free for 1 h at 37°C. The concentration of total RNA samples was determined spectrophotometrically at a wavelength of 260 nm. The RNA integrity was verified after gel electrophoresis in 1% agarose containing 0.5 μg/ml ethidium bromide, irradiation with ultraviolet light. The quantification of the total RNA samples was made using the NanoDrop ND-2000 spectrophotometer apparatus (NanoDrop Products, Wilmington, DE, USA) where 1U A260 RNA corresponds to 40 μg/ml. Samples were only used free of contaminants (A260/A230 ~ 1.8) and protein (A260/A280 = 1.8–2.0). The integrity of total RNA was assessed by observing the proportion of bands related to 18S and 28S rRNA on agarose gel electrophoresis 1% stained with SYBR® Safe (Life technologies). To eliminate genomic DNA contamination of the samples, 1 μg of total RNA (8 μL) was incubated with 1 unit (1 μL) of DNase I / RNase Free—(Invitrogen, USA) in the presence of one solution containing 20 mM Tris HCl, pH 8.4 and 2 mM MgCl2 for 15 min at 37°C, followed by incubation at 65°C for 10 min to inactivate the DNAse I.

After the above treatment, reverse transcription reaction was performed (RT-PCR) for cDNA synthesis. At 1 μg total RNA treated were added 2 μL of incubation buffer (50 mM KCl, Tris-HCl pH 8.4, 20 mM MgCl2, 2.5 mM), 1 unit of reverse transcriptase (1 μl) (Invitrogen) 2 μl Randon Primer (Invitrogen) 0.8 μL of oligonucleotides (dNTPs, 100 mM) and 4.2 μL of ultrapure H2O to a final reaction of 20 μL. The samples were then subjected to the following incubations: 25°C for 10 min, 37°C for 120 min, 85°C for 5 min. After the reaction, the cDNA samples were kept at −20°C for further Real-time PCR analysis.

The reaction of polymerization chain in real time (Real-Time PCR) combines PCR amplification with automated fluorescent detection. Amplification and data acquisition were performed with TaqMan probe using Abi Prism 7500 Fast equipment (Applied Biosystems) as previously described (Silva et al., 2014). The fluorescence excitation capture was performed on each PCR amplification cycle, providing a real-time quantification of the sequences of genes of interest. The protocol used for the Real time PCR reactions was: 1.0 μl of cDNA was added 5 μL of Solution TaqMan Fast Universal Master Mix 2X (Applied Biosystems, USA) and sufficient water to 10 μL reaction in each well of the 96 well-plate. The samples were applied in duplicate, and then incubated at 95°C for 20 s, and passed through 40 thermal cycles at 95°C for 3 s, 60°C for 30 s. TaqMan Array plates 96—Well-Plate FAST been customized by Applied Biosystems/Life Technologies according to the chosen genes.

All reactions were subjected to the same conditions of analysis and normalized by ROX passive reference dye signal for correction of fluctuations in reading due to changes in volume and evaporation over the reaction. The results, expressed in Ct value refer to the number of PCR cycles required for the fluorescent signal reaches the detection threshold. The differentially expressed genes were normalized by the expression level of the housekeeping genes GAPDH or 18S subunit ribosomal RNA, which expression was shown to remain unchanged under experimental conditions. Fast SDS 1.4 software (Applied Biosystems) was used for data processing. The ΔCt values of the samples were determined by subtracting the average Ct value of the mRNA of the target gene from the average Ct value of the GAPDH housekeeping gene or 18S rRNA. The 2^−ΔΔCt^ parameter was used to express the relative expression data.

### NanoString nCounter assay for miRNA profiling

We profiled miRNAs using NanoString nCounter-miRNA expression analyses (NanoString Technologies, Seattle, Washington) that uses molecular barcodes and single-molecule imaging to detect and count RNAs without PCR amplification to analyze the global expression of miRNAs in the same total RNA samples used for mRNA quantification. Those samples were isolated from septum 1 week after both MI procedures of occlusion and ablation. The method quantifies 423 endogenous rat miRNAs (based in miRBase, version 17) of each septal specimen remote to MI area in two plates that accommodated the 24 samples (*n* = 8/group).

Raw data were processed using the NanoStringNorm R package (Waggott et al., 2012). The raw data were log2 transformed and normalized using the mean of the six positive controls, which were used to calculate a scaling factor in each column (lane/sample) as suggested by NanoString. The internal positive spike controls were present in each reaction to account for minor differences in hybridization, purification, or binding efficiencies. The data were further background corrected by subtracting the mean of the six negative controls followed by quantile normalization.

Total RNA (100 ng) was used as input for nCounter miRNA sample preparation reactions and the reactions were performed, as per the manufacturer's instructions (NanoString Technologies). Small RNA sample preparation involves the ligation of a specific DNA tag onto the 3′ end of each mature miRNA. These tags normalize the melting temperatures of the miRNAs and provide a unique identification for each miRNA species in the sample. Excess tags were then removed, and the resulting material was hybridized with a panel of miRNA: tag-specific nCounter capture and barcoded reporter probes. Hybridized probes were then purified and immobilized on a streptavidin-coated cartridge using the nCounter Prep Station (NanoString Technologies). Data collection was carried out on the nCounter Digital Analyzer (NanoString Technologies) following manufacturer's instructions to count individual fluorescent barcodes and quantify target RNA molecules present in each sample. For each assay, a high-density scan (600 fields of view) was performed.

The nCounter miRNA data was also confirmed through cross-platform validation in 10 randomly selected study samples using the TaqMan probes on the 7500 Fast Real Time PCR System. Furthermore, differentially expressed miRNAs were also quantified by Real-time PCR (data not shown). The average Pearson correlation coefficient was 0.70 (0.60–0.80) between the two platforms, thus confirming the robustness of the nCounter platform.

### Statistical analysis

Data were analyzed with GraphPad Prism software (La Jolla, CA, USA). The Shapiro-Wilk test was used to verify normality and error variances. Results were evaluated using two-way ANOVA complemented by Tukey test was used to detect differences between three groups at sample with normal distribution. A *p* ≤ 0.05 was considered significant and the results are expressed as mean ± standard error of the mean (SEM).

## Results

### Mortality rate comparison

Twelve of 37 rats (32%) died post occlusion-induced MI, mainly because large infarction sizes. In contrast, only one rat died after RF-Ab of unspecific cause. This animal showed the commitment of 42% of the LV area, dismissing the idea of extensive Ml above average as a cause of death.

### TaqMan microrrays assays

Addressing to changes in remote MI mRNA expression after coronary occlusion compared to control, we observed 38 differentially expressed mRNAs involved in extracellular matrix remodeling [collagen III 1a (COLIII1a):1.59-fold; collagen I 1a (COLI1a): 3.09-fold; tenascin: 3.32-fold and matrix metallopeptidase 9: 2.41-fold], inflammation mediators [interleukin 6 (IL6): 1.06-fold; tumor necrosis factor receptor 1a (TNFRSFLA): 1.40-fold and tumor necrosis factor, alpha (TNFa): 1.13-fold] and a survival marker (AKT1: 1.65-fold) that were found to be upregulated after coronary occlusion compared to control.

Genes involved in apoptosis signaling [Bcl2 associated X protein (Bax): 1.10-fold and p 53: 1.11-fold] were found to be upregulated, whereas mitogem activated protein kinase 14 (MAPK14) and mitogem activated protein kinase 1 (MAPK1) were found to be downregulated (−1.07- and −1.09-fold, respectively) after coronary occlusion compared to control. Among oxidative stress genes, two were found to be upregulated after coronary occlusion [glutathione peroxidase 4 (GPX4): 1.34-fold and heat shock protein 12b (HSPA12B): 1.34-fold], whereas catalase and superoxide dismutase 1 were found to be downregulated by −1.03- and −1.04-fold, respectively.

Expressions of hypertrophy biomarkers [endothelin: 1.78-fold; natriuretic peptide A and B: 4.48- and 2.18-fold, respectively; protein kinase C, alpha: 1.13-fold and nuclear factor of activate T-cells cytoplasmic, calcineurin—dependent 3 (NFATC3): 1.10-fold] were found to be upregulated after coronary occlusion compared to control. Interestingly, calcineurin like EF hand protein 2, angiotensin converting enzyme (ACE1) and angiotensin II receptor type 1a (−1.77-, −1.05- and −1.19-fold, respectively) were found to be downregulated after coronary occlusion.

Two myofilamentar protein expressions were analyzed, myosin, heavy chain beta was found to be upregulated (1.59-fold) whereas myosin, heavy chain alpha was found to be downregulated (−1.52-fold) compared to control. The genes involved in calcium kinetics [phospholamban: 1.10-fold and solute carrier family 8 (sodium/calcium exchange), member 1: 1.38-fold] were found to be upregulated after coronary occlusion compared to control. However, also involved in calcium kinetics, ryanodine receptor 2, ATPase, calcium transporting cardiac muscle, slow twitch 2 (ATP2A2) and calsequestrin 2 (−1.25, −1.06-, −1.05-fold, respectively) were found to be downregulated after MI occlusion-induced. Vascular endothelial growth factor A (VEGFA, −1.50-fold) was found to be downregulated after occlusion when compared to control.

Among metabolism-related genes [hexoquinase I: 1.59-fold; uncopling protein 2: 1.56-fold; solute carrier family 2 (facilited glucose transporter) member 1: 1.08-fold and taffazin: 1.10-fold] were found to be upregulated after coronary occlusion. Apart from these, phosphofructokinase expression (−1.53-fold) was found to be downregulated after coronary occlusion compared to control.

Comparing remote MI mRNA expression after ablation protocol to control, we found 38 differentially expressed genes. Extracellular matrix remodeling genes were found to be upregulated [collagen III 1a (COLIII1a):1.65-fold; collagen I 1a (COLI1a): 3.87-fold; tenascin: 6.68-fold and transforming growth factor, beta 1 (TGF1b): 1.57-fold] when compared to control. Also, inflammation mediators genes [interleukin 6 (IL6): 1.17-fold and tumor necrosis factor, alpha (TNFa): 1.83-fold] showed increased expression after myocardial ablation in comparison with control.

Furthermore, mRNA expressions of apoptosis signaling genes [Bcl2 associated × protein (Bax): 1.30-fold, p 53: 1.82-fold, mitogem activated protein kinase 1 (MAPK1): 1.92-fold], survival effector (AKT1): 2.25-fold, calcium kinetics [ryanodine receptor 2: 2.29-fold; ATPase, calcium transporting cardiac muscle, slow twitch 2 (ATP2A2): 1.92-fold; calsequestrin 2: 1.93-fold; phospholamban: 3.36-fold and solute carrier family 8 (sodium/calcium exchange), member 1: 3.29-fold], metabolism-related genes [hexoquinase I: 2.74-fold; uncopling protein 2: 2.35-fold; solute carrier family 2 (facilited glucose transporter) member 1: 1.90-fold; phosphofructokinase: 1.18-fold; NADH dehydrogenase (ubiquinone) 1 alpha subcomplex 3 (NDUFA3): 2.27-fold and taffazin: 1.58-fold] were also found to be upregulated after ablation. In addition, myosin heavy chain beta (2.73-fold)] was found to be upregulated after ablation compared to control, however, the expression of the alpha isoform remained unchanged after ablation.

Among oxidative stress genes analyzed, two were found to be upregulated after ablation [glutathione peroxidase 4 (GPX4): 2.27-fold and catalase: 2.29-fold], whereas superoxide dismutase 1 was found to be downregulated by −1.22-fold when compared to control. In addition, expression of vascular endothelial growth factor A (VEGFA, −1.50-fold) was found to be downregulated after ablation when compared to control.

Most of the hypertrophy biomarkers [angiotensin converting enzyme (ACE1): 1.12-fold; angiotensin converting enzyme 2 (ACE2): 1.58-fold; angiotensin II receptor type 1a: 1.69-fold; endothelin: 2.22-fold; natriuretic peptide A and B: 1.91- and 2.21-fold, respectively; protein kinase C, alpha: 2.29-fold; nuclear factor of activate T-cells cytoplasmic, calcineurin—dependent 3 (NFATC3): 2.22-fold; insulin like growth factor (IGF1): 1.55-fold] were found to be upregulated after ablation compared to control. Among these protein kinase C, beta (−1.25-fold) was found to be downregulated after ablation.

Analyzing changes in cardiac mRNA expression in occlusion compared to ablation, we observed a smaller number of differentially expressed mRNA (nineteen genes) that were found to be differently expressed after occlusion than when comparison was performed against control). Genes involved in extracellular matrix remodeling [collagen III 1a (COLIII1a):1.25-fold; tenascin: 2.01-fold; matrix metallopeptidase 9: 2.03-fold and transforming growth factor, beta 1 (TGF1b): 1.42-fold], survival marker (AKT1: 1.37-fold), apoptosis signaling [mitogem activated protein kinase 14 (MAPK14): 1.21-fold], calcium kinetics [phospholamban: 6.16-fold; solute carrier family 8 (sodium/calcium exchange), member 1 (SLC8A1): 2.39-fold; calsequestrin 2: 2.04-fold and ATPase, calcium transporting cardiac muscle, slow twitch 2 (ATP2A2): 2.05-fold], metabolism-related genes [hexoquinase I: 1.72-fold; uncopling protein 2: 1.51-fold; solute carrier family 2 (facilited glucose transporter) member 1: 1.08-fold and taffazin: 1.75-fold and phosphofructokinase: 1.80-fold] were found to be upregulated after coronary occlusion when compared to ablation protocol.

None oxidative stress genes were differently expressed comparing both MI procedures. Regarding to inflammatory response genes, only tumor necrosis factor receptor, member 1a mRNA (TNFRSFLA, −1.05-fold) was found to be downregulated in occlusion when compared to ablation. Expressions of hypertrophy biomarkers [calcineurin like EF hand protein 2: 1.72-fold; angiotensin converting enzyme 2 (ACE2): 1.99-fold and insulin like growth factor 1 (IGF1):1.68-fold] were found to be upregulated after occlusion compared to ablation. Protein kinase C, gamma (−1.23-fold) was found to be downregulated after occlusion when compared to ablation. All data referring to Taqman Realtime PCR are summarized on Table 1.

**Table 1:** **Altered significantly mRNA content (***p*** ≤ 0.05) after myocardial infarction**.

**Gene ID**	**mRNA description**	**OC × SH**	**AB × SH**	**OC × AB**
		**Log2 fold**	**Log2 fold**	**Log2 fold**
**EXTRACELLULAR MATRIX**				
84032	COL3A1	1.60^*^	1.65^*^	unaltered
29393	COL1A1	3.10^***^	3.90^***^	1.25^***^
59086	TGF1B	unaltered	1.58^*^	1.42^*^
116640	TNC	3.32^***^	6.68^***^	2.01^*^
81687	MMP9	2.41^***^	unaltered	2.03^***^
**INFLAMMATION**				
24498	IL6	1.07^***^	1.18^*^	unaltered
25625	TNFRSFLA	1.41^*^	unaltered	−1.05^*^
24835	TNFa	1.13^*^	1.83^***^	unaltered
**APOPTOSIS**				
24887	BAX	1.11^*^	1.31^*^	unaltered
24842	TP53	1.11^*^	1.82^***^	unaltered
81694	MAPK14	−1.07^***^	unaltered	1.21^*^
116590	MAPK1	−1.09^***^	1.92^***^	unaltered
**OXIDATIVE STRESS**				
29323	GPX4	1.35^*^	2.28^*^	unaltered
24248	CAT	−1.04^***^	2.29^***^	unaltered
311427	HSPA12B	1.34^*^	unaltered	unaltered
24786	SOD1	−1.05^***^	−1.23^*^	unaltered
**ANGIOGENESIS**				
11651	AKT1	1.65^***^	2.26^*^	1.37^***^
83785	VEGFA	−1.50^***^	−1.51^*^	unaltered
**HYPERTROPHY**				
24310	ECA	−1.06^*^	1.13^*^	unaltered
302668	ECA2	unaltered	1.58^*^	1.99^***^
24323	EDN1	1.78^*^	2.22^***^	unaltered
24602	NPPA	4.49^*^	1.91^*^	unaltered
25105	NPPB	2.18^***^	2.22^*^	unaltered
24482	IGF1	unaltered	1.68^*^	1.68^*^
24681	PRKCG	unaltered	unaltered	−1.23^***^
24680	PRKCA	1.13^*^	2.29^***^	unaltered
308965	CHP2	−1.78^*^	unaltered	1.72^*^
24180	AGTR1A	−1.20^***^	1.70^***^	unaltered
361400	NFATC3	1.10^***^	2.23^***^	unaltered
**CELL SURVIVAL**				
29556	MYH6	−1.52^*^	2.73^*^	unaltered
29557	MYH7	1.60^***^	unaltered	unaltered
**CALCIUM SIGNALING**				
689560	RYR2	−1.30^*^	2.30^***^	unaltered
29693	ATP2A2	−1.07^***^	1.92^*^	2.05^*^
29209	CASQ2	−1.05^***^	1.94^***^	2.04^***^
64672	PLN	1.11^***^	3.36^***^	6.16^***^
29715	SLC8A1	1.38^***^	3.29^***^	2.39^*^
**METABOLISM**				
25058	HK1	1.59^***^	2.75^***^	1.72^*^
54315	UCP2	1.56^***^	2.36^*^	1.51^*^
24778	SLC2A1	1.09^***^	1.90^*^	1.75^***^
65152	PFKM	−1.54^***^	1.19^*^	1.80^***^
691001	NDUFA3	1.13^***^	2.27^***^	unaltered
363521	TAZ	1.10^***^	1.59^*^	unaltered

Gene ID refers to NCBI database numbers. OC × SH refers to Occlusion group vs. Sham animals, AB × SH refers to Ablation group compared to Sham animals, OC × AB refers to the comparison of both MI protocols.

* p ≤ 0.05,

*** p ≤ 0.001, unaltered refers to p > 0.05. More details are provided in Methods Section.

### High throughput miRNA analysis

From 423 miRNAs, we only found eight differentially expressed miRNAs among experimental groups. This low detection support the idea that, besides particular differences in miRNA expression between occlusion and ablation procedures, only few miRNAs showed differentially expressed among total miRNA (<2% of total miRNA). Following, we highlighted the differentially expressed miRNAs detected on NanoString platform.

The data show that mir-221 presented a method-specific switch in expression; occlusion had higher levels of these miRNAs than ablation. Similarly, miR-34c and mir-93 expressions were higher expressed in occlusion at 1 week compared to control, and also in occlusion compared to ablation.

We also identified miRNAs with changes in expression only after occlusion. Specifically, mir-301 and mir-17-5p showed different expression levels after occlusion, while mir-301 expression decreased, we observed an increased in mir-17-5p expression compared to control.

The mir-9 expression detected was similar between occlusion and ablation and different when compared to control, whereas both diminished. Ablation showed diminished expressions of mir-542-5p and mir-1949 compared to control and occlusion. Together, these results show that there are few changes in miRNAs content between MI methods 1 week after infarction. All data regarding to miRNA quantification are presented on Tables 2–4.

**Table 2:** **MiRNAs that were altered significantly (***p*** ≤ 0.05) after myocardial infarction induced by occlusion compared to Sham**.

**Column ID**	***p*-value (OC vs. SH)**	**Mean ratio (OC vs. SH)**	**Fold Change (OC vs. SH)**
rno-miR-221	0.000626783	6.98409	6.98409
rno-miR-301b	0.0350184	0.49866	−2.00537
rno-miR-34c	0.0415997	2.63543	2.63543
rno-miR-93	0.00615468	1.82695	1.82695
rno-miR-9	0.024583	0.328826	−3.04112
rno-miR-17-5p	0.047911	1.74863	1.74863

**Table 3:** **MiRNAs that were altered significantly (***p*** ≤ 0.05) after myocardial infarction induced by ablation compared to Sham**.

**Column ID**	***p*-value (AB vs. SH)**	**Mean Ratio (AB vs. SH)**	**Fold Change (AB vs. SH)**
rno-miR-1949	0.0117833	0.231907	−4.31208
rno-miR-542-5p	0.00367377	0.544582	−1.83627
rno-miR-9	0.0464964	0.414557	−2.41221

**Table 4:** **MiRNAs that were altered significantly (***p*** ≤ 0.05) after myocardial infarction induced by occlusion compared to ablation**.

**Column ID**	***p*-value (AB vs. OC)**	**Mean ratio (AB vs. OC)**	**Fold change (AB vs. OC)**
rno-miR-1949	0.0350587	0.270379	−3.69851
rno-miR-221	0.00071974	0.155874	−6.41545
rno-miR-542-5p	0.0117105	0.586736	−1.70435
rno-miR-34c	0.0157985	0.247443	−4.04134
rno-miR-93	0.0027246	0.494091	−2.02392

## Discussion

Data from transcriptomes have opened a window for more integrated analysis of both qualitative and quantitative changes in global mRNA expression at a particular time point of any biological system (Agnetti et al., 2007). In the last decade, there have been several efforts to dissect out cardiac mRNA and proteome in a wide array of cardiovascular diseases (McGregor and Dunn, 2006) using different animal models or plasma samples collected from human patients (Seenarain et al., 2010; Haas et al., 2011; Silbiger et al., 2011; Drastichova et al., 2012; Chowdhury et al., 2013; Marshall et al., 2014; Petriz and Franco, 2014). While novel biomarkers were identified from some of these studies (Haas et al., 2011; Silbiger et al., 2011; Chowdhury et al., 2013), authors reported general alterations in the cardiac transcriptome and proteome profile that suggested changes in Ca2+ handling proteins (Seenarain et al., 2010), energy metabolism proteins (Jin et al., 2006; Meng et al., 2009), and mediators of apoptotic signaling (Drastichova et al., 2012; Marshall et al., 2014; Petriz and Franco, 2014).

Here, we attempted to achieve differentially expressed mRNA of remote area of MI between coronary occlusion and by radio frequency ablation in rats. Heretofore, we believe our study is the first report that compares differentially mRNA expression profile and large-scale miRNA analysis of these different MI procedures, in order to establish ablation-induced MI as an alternative experimental MI method, with the advantages of uniformity of MI sizes and low mortality rates. Surprisingly, we observed more mRNA expression similarities than differences between these protocols.

Extracellular matrix (ECM) components are important for mechanical support and effective functioning of the cardiovascular system. Therefore, alteration of the ECM may directly result in changes of mechanical properties and functional impairment. The ECM also plays significant roles in tissue remodeling in stress responses. Several extracellular matrix genes take part of the cardiac remodeling after MI. In tune with earlier reports (Roy et al., 2006; Manchini et al., 2014), mRNA modifications regarding to cardiac remodeling were observed in our experimental groups. mRNA expression analysis revealed significant upregulation of most genes after both occlusion and ablation compared to control. We found an increased tenascin (TNC) mRNA expression in both occlusion and ablation. Tenascin is sparsely detected in the normal adult myocardium, but reappears when the heart remodels its structure in response to pathologic insults, such as acute MI (Willems et al., 1996; Imanaka-Yoshida et al., 2001; Sato et al., 2006; Odaka et al., 2008), myocarditis (Imanaka-Yoshida et al., 2002; Sato et al., 2002; Morimoto et al., 2005), hibernation (Frangogiannis et al., 2002), ischemia-reperfusion (Taki et al., 2010), hypertensive cardiac fibrosis (Nishioka et al., 2007), chronic cardiac rejection (Franz et al., 2010), and some cases of dilated cardiomyopathy (DCM) (Tamura et al., 1996; Tsukada et al., 2009) closely associated with inflammation. Several studies suggest that TNC could help tissue reconstruction of the edge of the residual myocardium as a de-adhesion protein. TNC could loosen strong adhesion of cardiomyocytes (Imanaka-Yoshida et al., 2001, 2004) and upregulate the expression and activity of matrix metalloproteinases (MMPs) (Collins et al., 2004). In fact, expression of matrix metalloproteinase 9 was found to be induced after occlusion but remained unaltered after ablation, indicating that this particular increased expression may be consequent to TNC stimulation. Most matrix cellular proteins are minimally expressed in normal young adult hearts, but are markedly upregulated following cardiac injury. Matrix cellular proteins induced in the infarcted heart appear to serve as transducers of key molecular signals in cardiac repair and act as modulators of cell migration, proliferation, and adhesion (Frangogiannis, 2012).

Fibrosis is often considered to be the end inflammatory reaction, and both stimuli generated by occlusion and ablation were able to increase collagen I mRNA content. Regarding to collagen III, the mRNA expression remained unchanged. This data is in consonance with other reports that indicated an augmentation in expression of collagen fibers type I as the end product when tissue is repaired during heart failure in animal models and human patients (Stefanon et al., 2013; Yabluchanskiy et al., 2013), while collagen III is quickly synthetized and gradually switched to collagen I fibers. A high expression of TGF1b in occlusion group compared to ablation suggested that there would be different pathways activated after this procedure. TGF-β1 is a persistent stimulus in the chronic and inappropriate wound healing phase that is marked by hypertrophic scarring and eventual stiffening of the entire myocardium, ultimately leading to the pathogenesis of heart failure following MI (Zeglinski et al., 2016). Moreover, TGFβ1 is a key pro-fibrotic cytokine that is markedly elevated in experimental MI, and anti-TGF gene therapy mitigates cardiac remodeling by affecting cardiac fibrosis and infarct tissue dynamics (Okada et al., 2005).

In the infarcted myocardium, necrotic cardiomyocytes release danger signals, activating an intense inflammatory response. Inflammatory pathways play a crucial role in regulation of a wide range of cellular processes involved in injury, repair, and remodeling of the infarcted heart. Pro-inflammatory cytokines, such as tumor necrosis factor α and interleukin 1, are markedly upregulated in the infarcted myocardium and promote adhesive interactions between endothelial cells and leukocytes by stimulating chemokine and adhesion molecule expression (Saxena et al., 2016).

In fact, we found that some genes involved in inflammation were upregulated after both occlusion and ablation. Upregulation of all these mRNAs after MI procedures could be due to an establishment of a secondary inflammation that accomplishes MI by necrosis and other cell death mechanisms as reported by earlier studies (Manchini et al., 2014; Saxena et al., 2016). Importantly, inflammation mediator expressions were mostly unaltered when occlusion and ablation were compared, indicating the same activation pathways, there was an increased mRNA expression of most interleukin analyzed in both procedures when compared to control. TNF receptor mRNA, for instance, showed increased expression in both procedures when compared to control, and presented a downregulation after occlusion when compared to ablation. This difference might indicate a variation in the time course and extension of stimulus that leads to inflammation. Several reports on the expression status of these mRNAs in occlusion-induced MI are available—and there is a consensus that inflammation plays an important role after cardiomyocytes death (Bao et al., 2008; Zhu et al., 2015).

Our study also revealed an upregulation of Bax and p53 mRNA after both procedures compared to control, although both genes remained unaltered compared among procedures, corroborating that the MI protocols show the same mRNA expression pattern. It has been reported earlier that MI presented an important upregulation of mitochondria-related pro-apoptotic members to meet increased stimulation after MI procedures (Xu et al., 2015). In occlusion, we observed a downregulation of mitogen activated protein kinases 1 and 14 when compared to control. These two kinases have several functions, while MAPK1 act as an integration point for multiple biochemical signals, and are involved in a wide variety of cellular processes such as proliferation, differentiation, transcription regulation and development, the MAPK14 is member of p38 MAPK family, responsive to stress stimuli, such as cytokines, ultraviolet irradiation, heat shock, and osmotic shock, and are involved in cell apoptosis, differentiation and autophagy. These intracellular mitogen-activated protein kinases (MAPKs) signaling cascades play a key role in the pathogenesis of cardiac and vascular disease. ERK1/2, a member of the MAPKs, is concerned with the regulation of cell proliferation, differentiation, survival, and apoptosis. Although, most researchers have maintained that the phosphorylation of ERK1/2 played an important role in protecting against myocardial I/R injury (Clark et al., 2007), others have shown that ERK1/2 phosphorylation may aggravate myocardial cell injury (El-Mahdy et al., 2016). There is strong *in vivo* evidence that activation of the p38 mitogen-activated protein kinase (MAPK) family of stress-activated kinases exacerbates myocardial injury following prolonged ischemia (Clark et al., 2007). Pro-inflammatory cytokines such as IL-1 and tumor necrosis factor alpha (TNFα), which are potent stimuli for the p38 MAPK pathway, are elevated in the infarcted heart and appear to be detrimental for post-MI myocardial remodeling and progression to heart failure (Aukrust et al., 2005).

Oxidative stress-related mRNA presented markedly expression in occlusion and ablation when compared to control, although showed similar expression in comparison between MI procedures. Reactive oxygen species (ROS) such as superoxide anions (·${\text{O}}_{2}^{-}$) and hydroxyl radicals (·OH) cause the oxidation of membrane phospholipids, proteins, and DNA (McCord, 1985) and have been implicated in a wide range of pathological conditions including ischemia-reperfusion injury (Chen et al., 2001), neurodegenerative diseases (Mizuno et al., 1998) and aging (Trifunovic et al., 2004).

Under physiological conditions, their toxic effects can be prevented by scavenging enzymes such as superoxide dismutase (SOD), mitochondrial phospholipid hydroperoxide glutathione peroxidase (GPX4), and catalase as well as by other non-enzymatic antioxidants. However, when the production of ROS exceeds the capacity of antioxidant defenses, oxidative stress might have a harmful effect on the functional and structural integrity of biological tissue. ROS cause contractile failure and structural damage in the myocardium (Toussaint et al., 1993). The importance of oxidative stress is increasingly emerging with respect to a pathophysiological mechanism of LV remodeling responsible for heart failure progression.

We identified GPX4 as the only oxidative stress molecule that showed increased mRNA expression after both procedures compared to control. GPX gene overexpression inhibited the development of LV remodeling and failure after MI, which might contribute to the improved survival (Shiomi et al., 2004). These findings not only extended the previous observation that employed antioxidants, but also revealed the major role of ROS in the pathophysiology of myocardial remodeling. These effects were associated with the attenuation of myocyte hypertrophy, apoptosis, and interstitial fibrosis (Shiomi et al., 2004). Similarly, overexpression of the GPX gene attenuated myocardial remodeling and preserved diastolic function in diabetic heart (Matsushima et al., 2006). Therefore, therapies designed to interfere with oxidative stress by using GPX could be beneficial to prevent myocardial remodeling and failure.

Superoxide dismutase 1 is the primary mitochondrial antioxidant enzyme and is essential for maintaining normal cell development and function. Overexpression of the SOD1 gene has been shown to be beneficial in various animal models of cardiac diseases (Yen et al., 1996; Chen et al., 1998). Interestingly, we found to be a downregulation of SOD1 mRNA after MI procedures compared to control. SOD1 gene overexpression also elevated levels of myocyte catalase and mitochondrial GSH, which might also act together with SOD1 against oxidative stress (Khaper et al., 2003). Catalase gene was differently expressed in each procedure compared to control. The expression increase in ablation could be related to the lesion extension after MI induction (Tsutsui et al., 2009).

Upregulation of hypertrophic markers after MI suggests an activation of a mechanism that promotes enlargement of the heart chambers as a compensative strategy to pathophysiologic state. Hence, it could be suggested that differences concerning hypertrophy in ablation when compared to control is an indicative of severity of stimulus triggered by this MI procedure (Zannad et al., 2010; Gaggin and Januzzi, 2013). Ligation of coronary artery provokes an augmentation on hypertrophic markers, such as the cardiac natriuretic peptide A content, that showed significant upregulation when compared to ablation. The natriuretic peptides represent the gold standard for biomarkers in HF, and the understanding about their biology and their clinical use have both grown exponentially since their introduction. Structurally conserved across multiple species, a number of structurally similar natriuretic peptides have been identified: atrial natriuretic peptide (ANP), urodilantin (an isoform of ANP), B-type natriuretic peptide (BNP), C-type natriuretic peptide and Dendroaspis natriuretic peptide (Cea, 2005). Of these, ANP and BNP are transcribed and primarily produced in the myocytes of atria and ventricles, respectively (Mukoyama et al., 1991), both are produced in response to myocardial stretch due to pressure or volume overload (Kinnunen et al., 1993), conditions commonly found in HF. The biological functions of ANP and BNP include various compensatory mechanisms such as natriuresis, diuresis, and vasodilation (Cody et al., 1986; Marcus et al., 1996).

Myofilamentar fibers also are important markers of hypertrophy. Myosin isoform beta was upregulated after MI procedures compared to control, which is in agreement to previous report where myosin over-expression is related as a molecular sign of overloaded myocardium (Gupta, 2007). Although an augmentation of mRNA expression was observed when experimental groups were compared to control, there were no expression changes between both MI procedures. A major shift in the myosin isoform distribution occurs during pathologic hypertrophy mediated by pressure and volume overload. This hypertrophy is associated with induction of βMHC at the expense of αMHC. This change from the α- to β-MHC phenotype is taken as a hallmark of pathologic hypertrophy, which is more intense in pressure-overload than in volume-overload hypertrophy (Gupta, 2007).

Particularly, all five genes related to calcium dynamic showed altered mRNA expression in our study. Drastic increase in the level of phospholamban and sodium calcium exchange SLC8A1 mRNAs were observed after MI generated by ablation compared to both occlusion and control. Failing heart muscle generally exhibits distinct changes in intracellular calcium (Ca^2+^) handling, including impaired removal of cytosolic Ca^2+^; reduced Ca^2+^ loading of the cardiac sarcoplasmic reticulum (SR) with downregulation of SR Ca^2+^-ATPase 2 (SERCA2); and defects in SR Ca^2+^ release accompanied by impairment of cardiac relaxation and systolic function (Morgan et al., 1990; Marx et al., 2000). Sodium-calcium exchange (NCX) is the major Ca^2+^ efflux mechanism of ventricular cardiomyocytes. Consequently, the exchanger plays a critical role in the regulation of cellular Ca^2+^ content and hence contractility (Ottolia et al., 2013).

On the other hand, calsequestrin 2, cardiac ryanodine receptor and ATPase, calcium transporter were downregulated after coronary occlusion compared to control. Calsequestrin (CSQ2), as the major Ca^2+^ binding protein in the sarcoplasmic reticulum of cardiac myocytes, communicates changes in the luminal Ca^2+^ concentration to the cardiac ryanodine receptor (RYR2) channel (Gaburjakova et al., 2013). Combined with biochemical observations highlighting the Ca^2+^ dependence of the CSQ2-RYR2 interaction, these results indicate that the changes in RYR2 activity caused by luminal Ca^2+^ may be presumably attributed to the CSQ2 dissociation from the channel complex; and thus, it is highly likely that CSQ2 plays an active role in communicating changes in [Ca^2+^] to the RYR2 channel (Gaburjakova et al., 2013).

The metabolic adaptation of heart glycolysis to substrate availability, workload, and hormones has been known for many years (Taegtmeyer, 1994; Stanley et al., 1997) and the clinical relevance of glucose metabolism to heart diseases has been reviewed (Taegtmeyer, 1994; Depré et al., 1998). Six mRNA implied to the metabolism of cardiomyocytes were relevant in our expression investigation. All six genes were found to be upregulated after MI induced by occlusion and ablation compared to control, a common expression pattern seem before in our mRNA quantification. Phosphosfructokinase 1, a tetrameric enzyme that phosphorylates fructose-6-phosphate to fructose-1,6-bisphosphate, committing glucose to glycolysis (Opie, 1978), was decreased after occlusion, despite the fact that it was more marked after ablation, both compared to control. An augmentation of phosphofructokinase mRNA in occlusion compared to ablation might indicate a more intense metabolic rate after hypoxia, to reverse cell damage and death.

Two detected mRNA in this group, NAD dehydrogenase 1 alpha 3 and taffazzin (TAZ), increased after both MI procedures compared to control. So far, there are no clear reports on taffazin expression; while some authors observed a remarkable over 90% decrease in TAZ mRNA expression during the development of HF in rats and humans, others reported that its expression remains unchanged after MI (Athéa et al., 2007; Harjot et al., 2009). The NADPH oxidase family (Nox) is a major and dedicated cellular ROS generation system in cardiac myocytes and many other cell types, including neurons (Lambeth, 2004; Bedard and Krause, 2007). It has been suggested that decreases in NAD^+^/NADH ratio, which have been observed in mitochondrial diseases or metabolic diseases such as diabetes, promote reductive stress (Ido, 2007; Ying, 2008). Increased NADPH level facilitates the reduction of glutathione, which serves as an important antioxidant mechanism. It should be mentioned that ischemia elevates NADH level (NAD^+^/NADH ratio declines; Ying, 2008).

VEGFa, usually referred to as VEGF, acts as a key player in vasculogenesis and angiogenesis. VEGFa mRNA was found to be downregulated after both MI procedures compared to control. Zhao et al. (2010) found that VEGFa levels within the infarcted myocardium were persistently suppressed post MI. VEGFR expression was significantly increased only at the border zone at day 1, but not in the later stages. The expression of VEGFa/VEGFR remained unchanged in the noninfarcted myocardium.

On the other hand, Akt1 was upregulated exclusively after MI procedures compared to control. Multiple signaling pathways downstream of Akt1 control cell survival, growth, metabolism, cell cycle progression, as well as motility of vascular cells (Somanath et al., 2006). Akt1 signaling might be involved in the regulation of several aspects of cardiac function and repair following an ischemic injury. Akt1 signaling seems to promote fibrosis in post-MI hearts. Akt1 function in cardiac fibrosis and hypertrophy is supported by multiple studies using both overexpression of Akt1 in heart (Nagoshi et al., 2005; Shiojima et al., 2005; Shiojima and Walsh, 2006) and Akt1 deletion (Shimizu et al., 2010) models. Although lack of Akt1 enhances LV damage and apoptosis in cardiomyocytes immediately after I/R injury, in a longer term, it improves cardiac function and LV remodeling after MI (Shimizu et al., 2010).

### miRNA analysis

In the current study, we found only few microRNAs are differentially expressed between both MI models or compared to control. All differentially expressed miRNA expression were validated with Real time PCR and presented decreased expressions after ablation compared to occlusion as observed in nCounter analyzes. In response to ischemia or I/R, cardiac cells including cardiomyocytes, cardiac fibroblasts, and endothelial cells undergo variable miRNA dysregulation. These changes can be apparent as early as 15 min after coronary artery ligation and may last for hours, days, or months (Zhou et al., 2016). This dysregulation may comprise a mix of protective and deleterious effects. Following very brief ischemia or reperfusion, such changes in miRNA expression might be relevant to early cell survival. A prolonged ischemia and/or reperfusion will cause permanent injury to the heart, and changes in miRNA expression may be pertinent to apoptotic status and other chronic processes including cardiac fibrosis and remodeling (Zhou et al., 2016).

In our occlusion protocol, we observed an enhanced expression of miR-221. Notwithstanding, we detected an increased ANP and BNP mRNA expression levels after 1 week. These mRNA increments observed in MI generated by occlusion is well established. Upregulation of miRNA-221 is also related to antiapoptotic effects in cancer cells (Zhou et al., 2016) and showed pro-proliferative, pro-migration, and antiapoptotic effects in vascular smooth muscle cells (Zhou et al., 2016). The miRNA-221 is significantly upregulated in patients with hypertrophic cardiomyopathy (HCM) and in a mouse model of cardiac hypertrophy and heart failure induced by pressure overload (Su et al., 2015). *In vitro* overexpression of miR-221 alone is sufficient to increase the size of cardiomyocytes, accompanied by enhanced expression levels of atrial natriuretic peptide (ANP) and brain natriuretic peptide (BNP) (Wang et al., 2012). However, the *in vivo* roles and molecular mechanisms of miR-221 in the regulation of cardiac remodeling remain unclear.

Our study identified an upregulation of miR-34c after occlusion compared to control, and its upregulation compared to ablation. Reports showed that miR-34c is induced after acute ischemic damage (Greco et al., 2009), miR-34b and miR-34c belong to an evolutionary conserved miRNA family that plays a fundamental role in the p53 tumor suppressor network (He et al., 2007). Since MI procedures promotes p53-dependent apoptosis (Fiordaliso et al., 2001), it is tempting to speculate that the p53/miR-34 axis may be involved in a MI-induced death pathway.

Panguluri et al. (2013) identified a significant elevation of miR-301a as a key modulator in diabetic condition. Loss of function approach in *in vitro* model (H9C2 cells) using miR-301a inhibitor was used to test if the increase in miR-301 has a role in regulation of Kv4.2 gene expression, a voltage-gated K^+^ channel. We observed a decreased miR-301 and miR-93 expressions in occlusion group compared to ablation. Computational analysis of the *Homo sapiens* and *Mus musculus* RyR2-3′UTR revealed conserved binding sites for miR-93. These results demonstrate that miR-93 negatively regulates RyR2-3′UTR, which could lead to a decreased RyR2 protein expression (Chiang et al., 2014). In fact, we, analyzing downstream, observed a decrease in RyR2 mRNA expression in occlusion model compared to control.

MiR-17 was identified as a novel Apaf-1-targeting miRNA (Song et al., 2015). The delivery of exogenous miR-17 suppressed the apoptotic protease activating factor 1 (Apaf-1) expression and consequently attenuated formation of the apoptosome complex containing caspase-9, as demonstrated by co-immunoprecipitation and immunocytochemistry. Furthermore, miR-17 suppressed the cleavage of procaspase-9 and the subsequent activation of caspase-3, which is downstream of activated caspase-9. Together, these results demonstrated the potential of miR-17 as an effective anti-apoptotic agent (Song et al., 2015). We detected an increased expression of miR-17-5p only after occlusion procedure. Moreover, Li et al. (2013) investigated the role of miR-17 in cardiac matrix remodeling following myocardial infarction (MI). Using real-time PCR, miR-17 was up-regulated most dramatically: 3.7-fold and 2.4-fold in the infarct region 3 and 7 d post-MI, respectively, and 2.4-fold in the border zone at d 3 compared to sham control (*P* < 0.01). Thus, it suggests that miR-17 participates in the regulation of cardiac matrix remodeling and provides a novel therapeutic approach using miR-17 inhibitors to prevent remodeling and heart failure after MI (Li et al., 2013).

Wang et al. (2010) evidenced that miR-9 can suppress myocardin expression, a transcriptional cofactor of NFATc3 expressed at a relatively low level in cardiomyocytes under physiological conditions. However, it can be up-regulated by hypertrophic stimulation and consequently mediate hypertrophic signals. Administration of miR-9 could attenuate cardiac hypertrophy and ameliorate cardiac function. We observed a diminished miR-9 expression on both MI procedures compared to control, corroborating the data presented by Wang et al. (2010), whereas miR-9 suppression might increase cardiac hypertrophy by NFATc3 stimulation. In fact, we also observed an increased NFATc3 mRNA expression after occlusion and ablation 1 week-post MI compared to sham.

To the best of our knowledge, reports of miR-542 and miR-1949 expressions in health or damaged cardiac tissue were not yet described. We found a downregulation of both miRNAs in ablation compared to occlusion and control. MiR-542-5p may induce double-strand DNA breaks and reactive oxygen species accumulation in transfected cells (Faraonio et al., 2012). In addition, Yoon et al. (2010) suggested that survivin is a direct target of miR-542-3p and growth inhibition by miR-542-3p may have a potential utility as an anti-cancer therapy. The expression of miR-1949 was found to be deregulated and abundant in the rat bladder following spinal cord injury. Bioinformatics demonstrated that retinoblastoma 1, which is involved in tumorigenesis, is a target gene of miR-1949 (Wang et al., 2015), but still there was not a clear relation to the myocardium.

## Conclusion

We have identified transcriptional modifications in remote area of rat myocardium after MI induced by occlusion and ablation. Our findings pointed out that myocardium reacts, besides different insults, with more similarities than differences in areas remote to MI. Cardiac mRNA signatures after MI generated by both protocols were found to cause alterations in almost the same genes. This indicates a pattern in the way the tissue deals with the injury, although the response magnitude varies from one procedure to another. Notwithstanding, few miRNAs were detected to be differently expressed between occlusion and ablation experimental groups. Evaluation of mRNA expression of an increased number of genes and proteome would lead to better understanding of the miRNA targets associated with pathophysiological states seen in both MI procedures. Considering all aspects addressed in this study, we compared a genic and post-transcriptional modifications signatures in both methods and concluded that RF-Ab might be an alternative protocol to achieve MI with lower rates of animals' mortality and with a simple and reproducible method and mostly importantly, with activation of resembling signal pathways. However, we hope to address to more detailed analyses and pathway intersections in others studies in current development in our laboratory.

## Ethics statement

Ethics Committee for Animal Care and Use at the Nine of July University, UNINOVE. Consent number 034/2012.

## Author contributions

JS: Idealization, execution, and writing of the manuscript. ES: Execution and analysis of the data. RF: Execution and analysis of the data. AS: Idealization and analysis of the data. EB: Execution and analysis of the data. EA: Execution of protocols. PT: Idealization and analysis of the data. LN: Idealization and analysis of the data. MM: Idealization and analysis of the data.

### Conflict of interest statement

The authors declare that the research was conducted in the absence of any commercial or financial relationships that could be construed as a potential conflict of interest.
